# A follow-up study of a randomized controlled study evaluating safety and efficacy of leuprorelin acetate every-3-month depot for 2 versus 3 or more years with tamoxifen for 5 years as adjuvant treatment in premenopausal patients with endocrine-responsive breast cancer

**DOI:** 10.1007/s12282-020-01205-w

**Published:** 2021-02-27

**Authors:** Junichi Kurebayashi, Eiichi Shiba, Tatsuya Toyama, Hiroshi Matsumoto, Minoru Okazaki, Tadashi Nomizu, Tohru Ohtake, Takaaki Fujii, Yasuo Ohashi

**Affiliations:** 1 Department of Breast and Thyroid Surgery, Kawasaki Medical School, 577 Matsushima, Kurashiki, Okayama 701-0192 Japan; 2 Department of Breast Surgery, Osaka Breast Clinic, 1-13-8 Ohiraki, Osaka Fukushima-ku, Osaka, 553-0007 Japan; 3 Department of Breast Surgery, Nagoya City University Graduate School of Medical Sciences, 1-Kawasumi, Mizuho-cho, Mizuho-ku, Nagoya-shi, Aichi, 467-8602 Japan; 4 Division of Breast Surgery, Saitama Cancer Center, 780 Komuro, Ina-machi, Kitaadachi-gun, Saitama, 362-0806 Japan; 5 Division of Breast Surgery, Sapporo Breast Surgical Clinic, 19-22-6 Kita 6-jonishi, Sapporo Chuo-ku, Hokkaido, 060-0006 Japan; 6 Department of Surgery, Hoshi General Hospital, 159-1 Mukaigawaramachi, Koriyama-shi, Fukushima, 963-8501 Japan; 7 Department of Breast Surgery, Fukushima Medical University School of Medicine, 1 Hikariga-oka, Fukushima-City, Fukushima 960-1295 Japan; 8 Division of Breast and Endocrine Surgery, Gunma University Hospital, 3-39-15 Showamachi, Maebashi-shi, Gunma, 371-8511 Japan; 9 Department of Integrated Science and Technology, Chuo University, 1-13-27 Kasuga, Bunkyo-ku, TokyoTokyo, 112-8551 Japan

**Keywords:** LH-RH agonist, Ovarian function suppression, Adjuvant endocrine therapy, Premenopausal patient, Endocrine-responsive breast cancer

## Abstract

**Background:**

Previously, we conducted the 5-year open-label, randomized controlled trial (RCT) of leuprorelin adjuvant therapy in post-operative premenopausal patients with endocrine-responsive breast cancer, which was a pilot study to investigate the optimal duration of leuprorelin treatment. Since, however, long-term outcomes became required for the adjuvant endocrine therapy, we performed this follow-up observation study.

**Methods:**

Follow-up observation study was performed up to 10th year after randomization, continuing RCT to evaluate the efficacy and safety of leuprorelin every 3 months for ≥ 3 versus 2 years, with daily tamoxifen for 5 years. Primary endpoints were disease-free survival (DFS) and 2-year landmark DFS.

**Results:**

Eligible patients (*N* = 222) were randomly assigned to receive leuprorelin for either 2 years (*N* = 112) or ≥ 3 years (*N* = 110) with tamoxifen. Leuprorelin treatment for ≥ 3 years versus 2 years provided no significant difference in DFS (HR 0.944, 95% CI 0.486–1.8392) or 2-year landmark DFS (*N* = 99 and 102 in 2-year and ≥ 3-year groups, HR 0.834, 0.397–1.753). In small, higher-risk subgroup (*n* = 17); however, 2-year landmark DFS in ≥ 3-year group was significantly longer (HR 0.095, 0.011–0.850) than that in 2-year group. The incidence of bone-related adverse events was around 5% in both groups.

**Conclusions:**

Adjuvant leuprorelin treatment for ≥ 3 years with tamoxifen only showed similar efficacy and safety profiles to those for 2 years in analyses among all patients but suggested greater benefit in higher-risk patients. No new safety signal was identified for long-term leuprorelin treatment.

**Trial registration number:**

Not applicable. This was an observational study.

## Introduction

Adjuvant treatment with tamoxifen has been established as a standard therapy for pre- and postmenopausal women with estrogen-receptor (ER)-positive early breast cancer [1–4]; however, it can stimulate pituitary-ovarian function, accompanied by increased serum estradiol (E2) levels [5]. Luteinizing hormone-releasing hormone (LH-RH) agonists are an effective adjuvant therapy for premenopausal women with endocrine-responsive breast cancer, which can downregulate follicle-stimulating hormone (FSH) and LH, resulting in suppression of ovarian function and serum E2 level [6–9]. LH-RH agonist is considered an ideal combination partner of tamoxifen. Based on the randomized clinical trials, which showed a significant survival benefit of the combination of tamoxifen plus LH-RH agonist [10–12], the combination has been used as a postoperative adjuvant therapy for premenopausal women with endocrine-responsive early breast cancer [13]. Several adverse effects related to ovarian function suppression (OFS) are known, with a decrease in bone mineral density (BMD) most common [14]. Although sufficient evidence concerning the duration of OFS with tamoxifen has not been accumulated, the combination of 5 years of tamoxifen plus 2–5 years of OFS with an LH-RH agonist has been used as a postoperative adjuvant therapy [15].

Recently, the results from two randomized trials, Suppression of Ovarian Function Trial (SOFT) and Tamoxifen and Exemestane Trial (TEXT), were reported and clarified that the addition of OFS to tamoxifen resulted in significantly higher 8-year rates of both disease-free and overall survival (OS) than tamoxifen alone in premenopausal women with breast cancer [16]. On the other hand, the frequency of adverse events (AEs) was higher in the OFS groups than in the tamoxifen-alone group. The guidelines have recommended 5 years of tamoxifen alone or combination with 5 years of OFS as a standard adjuvant therapy for higher-risk premenopausal patients with early breast cancer [17, 18]. Although SOFT and TEXT showed the superior efficacy of 5-year OFS and tamoxifen, the optimal duration of LH-RH agonists has not been elucidated yet. Additionally, Japanese patients were not involved in those studies. The safety profile of endocrine therapy should also consider the racial difference.

Leuprorelin acetate (leuprorelin), an LH-RH agonist, is available as depot formulations for subcutaneous administration every 1 or 3 months for the treatment of hormone-responsive cancers, such as prostate cancer [19] and premenopausal breast cancer [20–23].

To investigate efficacy, safety, and the appropriate treatment duration for leuprorelin in Japanese patients, we conducted an open-label, randomized-controlled pilot study comparing leuprorelin treatment every 3 months for 2 years versus 3 or more (≥ 3) years in combination with tamoxifen given daily for 5 years in patients with endocrine-responsive breast cancer [24]. However, the 5-year study period became recognized as too short to evaluate the efficacy of adjuvant endocrine therapy after the start of this randomized controlled trial (RCT) [25–29]. We, therefore, performed follow-up observation study (FOS), continuing the RCT up to the 10th year after the randomization, considering 10-year follow-up appropriate for a pilot study.

## Patients and methods

### Patients

The eligibility criteria of the RCT have been described previously [24]. Briefly, premenopausal patients with histologically confirmed primary breast cancer who met the following criteria were eligible: age ≥ 20 years; both or either ER-positive or progesterone receptor (PgR)-positive primary tumor; T1–T3, any N, and M0; any type of surgical procedure prior to enrollment; performance status 0 or 1.

Major exclusion criteria included the following conditions or situations: endocrine therapy prior to surgery; postoperative adjuvant endocrine therapy before enrollment; bilateral oophorectomy and irradiation to bilateral ovaries; inflammatory breast cancer or bilateral breast cancer; multiple cancers or history of carcinoma in other organs.

The eligible patients for the FOS were participants in the RCT, available to continue the study from 2 years (96 weeks) after their randomization, and who provided written informed consent (IC) for the FOS.

### Study design

The detailed study design of the RCT has been described in the previous report [24]. Eligible patients were randomly assigned at a 1:1 ratio to receive leuprorelin (11.25 mg) subcutaneous administration every-3-month depot either for 2 years or ≥ 3 years, up to 5 years, in combination with tamoxifen (20 mg daily) given orally for 5 years. Random assignment was performed using dynamic allocation with the number of positive axillary lymphnodes, tumor diameter, ER/PgR status, age, pre- and post-operative chemotherapy (presence, absence), and study site.

For the 3-or-more-year treatment group (≥ 3-year group), patients who completed the leuprorelin treatment for 3 years (144 weeks) could extend that treatment for up to 5 years (240 weeks in total) if they were considered appropriate for continuing the extension study with written IC, while tamoxifen was administered throughout the 5-year study period of the RCT. Patients were also allowed to receive anti-osteoporosis drugs as needed.

**Fig. 1:**
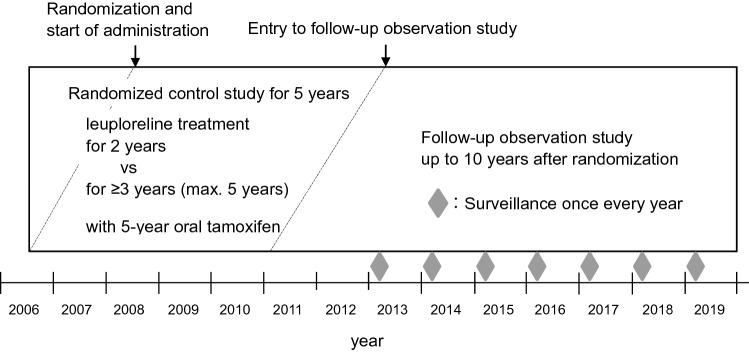
Study design and timeline

The FOS was started at the end of the 5-year study period in the RCT for each patient who provided new written IC during the RCT, and then follow-up activities were shifted from the RCT to the FOS (Fig. 1). A survey on the patients was conducted simultaneously in the all centers once every year up to the 10th year (the 520th week) from the initial administration. Matters for the survey were survival (or cause and date of death), recurrence (region, date, and diagnostic methods), secondary malignancies (same as recurrence), and bone-related AEs (osteoporosis or osteopenia, and bone fracture) including a confirmation date and way or procedure.

The RCT and FOS were conducted in accordance with the International Conference on Harmonisation of Good Clinical Practice Guidelines, the principles of the Declaration of Helsinki, and all applicable laws and regulations at 19 and 16 medical centers in Japan, respectively, between June 2006 and March 2019. The protocols of the RCT and FOS were reviewed and approved by the institutional review boards of all participating study sites. All patients provided written IC for participation before enrollment in both the RCT and FOS.

### Primary and secondary endpoints

Primary endpoints were disease-free survival (DFS), defined as the time from random assignment to disease event (recurrence, second primary cancer, or death), and 2-year (96-week) landmark DFS (DFS_L2y), defined as the time from the second year after randomization to disease event. The secondary endpoints were OS, defined as the time from randomization to death throughout the study period, and 2-year (96-week) landmark OS (OS_L2y), defined as the time from the second year after randomization to death. If the observation period ended before any disease event occurred, the DFS time was censored. Other measures included menstruation status, quality of life, and levels of E2, LH, and FSH throughout the 5-year RCT period.

Safety data were obtained throughout the 5-year RCT period, but were not collected in FOS except for the survey of bone-related events.

### Statistical analysis

The original statistical analysis plans for the RCT have been reported [24]. Since the RCT was a pilot study, the sample size was determined considering the feasibility, with a planned enrollment of 220 patients (110 per group). The patients were randomly assigned to two groups, and received leuprorelin for either 2 years or ≥ 3 years per group. DFS and OS were evaluated in the full analysis set (FAS), defined as data from the patients receiving at least one dose of the study drug after randomization throughout the study period of the RCT and FOS. DFS_L2y and OS_L2y were evaluated in a modified FAS (mFAS) containing data from all the patients who could continue the RCT after the second year (96th week) in the FAS.

Time-to-event methods (Kaplan–Meier and Cox proportional-hazards methods) were used to estimate the distributions of DFS, DFS_L2y, OS, and OS_L2y, and compare the 2-year and ≥ 3-year groups. The point estimates for each group, the differences between the groups, and their 2-sided 95% confidence intervals (CIs) were calculated at each timepoint according to Greenwood’s formula. To compare the survival curves (2-year versus ≥ 3-year groups), the log rank test was applied to test the null hypothesis of no difference, and the hazard ratios (HR, 95% CI) were estimated by applying the Cox proportional hazard regression model (Cox model). For statistical testing, the significance level was set at 0.05 (2-sided). Statistical multiplicity was not adjusted. All statistical analyses mentioned above were performed using SAS version 9.4 (SAS Institute, Cary, NC, USA).

Exploratory survival analyses were performed using the R statistical language (v3.5.3) and the R library (survival) [30, 31]. The interaction effect of risk (higher versus low risks) and treatment (≥ 3-year versus 2-year) on 2-year landmark DFS was analysed using multivariate Cox model in mFAS. Wald test was applied to test null hypothesis of no interaction. HR (95% CI) for DFS between two subgroups was estimated using univariate Cox model to evaluate risk factors. Odds ratios (OR, 95% CI) comparing incidence rates of events (≥ 3-year versus 2-year treatment groups) were calculated using the R library (Epi) [32].

## Results

### Patients

**Fig. 2:**
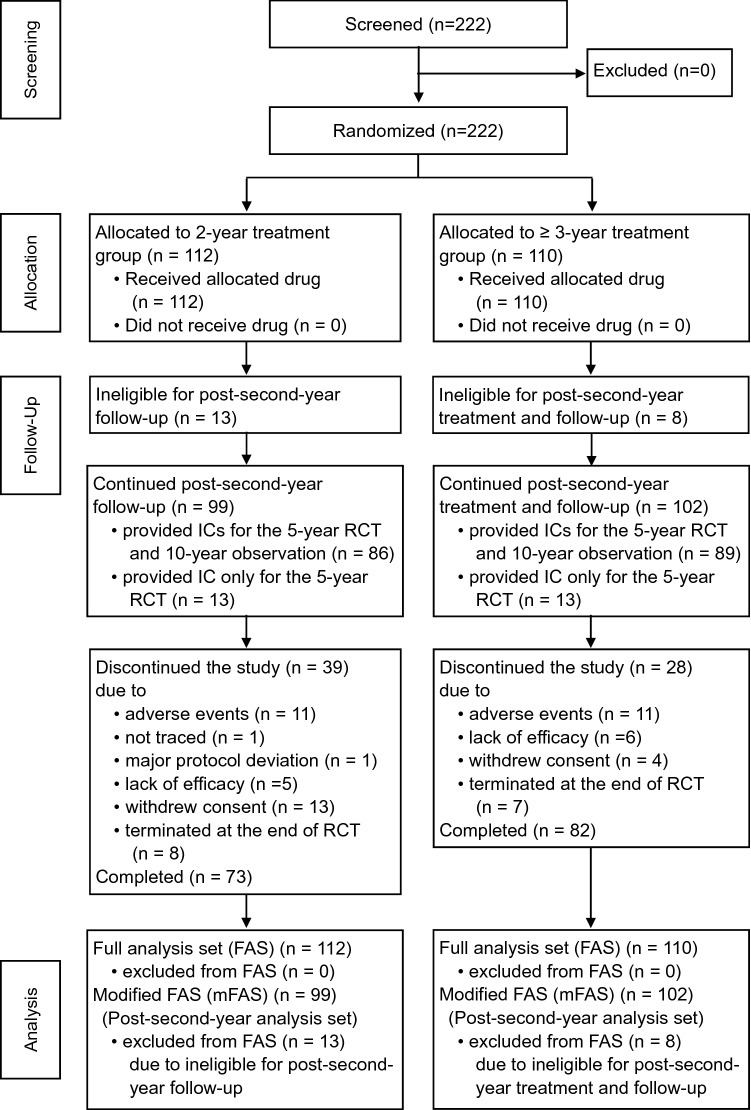
Patient disposition. *FAS* full analysis set, *IC* informed consent, *RCT* randomized controlled trial

The RCT with a 5-year study period was conducted at 19 sites in Japan, and 222 patients were enrolled between July 2006 and July 2008, randomly assigned to two groups, and received leuprorelin for either 2 years (*N* = 112) or ≥ 3 years (*N* = 110), respectively (Figs. 1 and 2). During the RCT, 186 patients (92 and 94 in 2-year and ≥ 3-year groups, respectively) were provided IC for the FOS and 175 patients (86 and 89) among them were available to continue from 2 years after the randomization. 36 patients (20 and 16) did not provide the IC for the FOS and 26 patients (13 patients in each group) among them who could continue the study after the 2nd year were handled as censored cases at the end of the RCT. Overall, 139 patients (68 and 71) completed the 10-year study period of the RCT and FOS, and 36 patients (16 and 22) discontinued the study (Fig. 2). All data from randomized patients (222) in the RCT were included in the FAS. For the 2-year landmark analyses, the mFAS comprised data of 201 patients (99 and 102 in 2-year and ≥ 3-year groups, respectively) whose post-second-year data were available for the analysis. There were 13 and 8 patients from the FAS who were ineligible for the mFAS, due to early termination before 2 years after the randomization for reasons as follows: adverse events, 4 and 4; lack of efficacy, 2 and 4; withdrawal of consent, 6 and 0; major protocol deviation, 1 and 0 in 2-year and ≥ 3-year groups, respectively.

Table 1 summarizes the baseline demographic and disease characteristics of patients in the mFAS. The baseline characteristics in the FAS have been reported [24]. There were no significant differences between the two groups except for serum E2 levels.

**Table 1:** Patient demographics and clinical characteristics in modified full analysis set

Baseline characteristics	Overall	2 years	≥ 3 years
	(*N* = 201)	(*N* = 99)	(*N* = 102)
Age (years)			
Median	44.0	44.0	43.5
Range	25.0–56.0	25.0–52.0	27.0–56.0
≤ 39 (%)	57 (28.4)	29 (29.3)	28 (27.5)
40–44 (%)	54 (26.9)	25 (25.3)	29 (28.4)
≥ 45 (%)	90 (44.8)	45 (45.5)	45 (44.1)
BMI (kg/m^2^)			
Mean (SD)	21.83 (3.50)	21.93 (3.45)	21.74 (3.55)
Tumor stage (TNM classification)			
I	135 (67.2)	68 (68.7)	67 (65.7)
IIA	56 (27.9)	26 (26.3)	30 (29.4)
IIB	9 (4.5)	4 (4.0)	5 (4.9)
IIIA	1 (0.5)	1 (1.0)	0 (0.0)
IIIB	0 (0.0)	0 (0.0)	0 (0.0)
Tumor size (cm)			
≤ 2	153 (76.1)	76 (76.8)	77 (75.5)
> 2	48 (23.9)	23 (23.2)	25 (24.5)
Number of axillary lymph nodes			
0	182 (90.5)	90 (90.9)	92 (90.2)
1–3	16 (8.0)	7 (7.1)	9 (8.8)
≥ 4	3 (1.5)	2 (2.0)	1 (1.0)
ER/PgR expression			
ER+/PgR+	188 (93.5)	90 (90.9)	98 (96.1)
ER+/PgR−	9 (4.5%)	5 (5.1)	4 (3.9)
ER−/PgR+	4 (2.0%)	4 (4.0)	0 (0.0)
Performance status (%)			
0	201 (100)	99 (100)	102 (100)
1–4	0 (0)	0 (0)	0 (0)
Preoperative chemotherapy			
Presence	1 (0.5)	1 (1.0)	0 (0.0)
Absence	200 (99.5)	98 (99.0)	102 (100.0)
Postoperative chemotherapy			
Presence	19 (9.5%)	10 (10.1)	9 (8.8)
Absence	182 (90.5)	89 (89.9)	93 (91.2)
Serum estradiol (pg/mL) at week 0			
Mean (SD)	129.1 (137.9)	104.6 (92.1)	152.9 (168.1)
Serum LH (mIU/mL) at week 0			
Mean (SD)	7.402 (9.965)	7.115 (8.154)	7.680 (11.488)
Serum FSH (mIU/mL) at week 0			
Mean (SD)	10.797 (14.183)	11.270 (14.549)	10.338 (13.875
Tumor marker/CEA (ng/mL)			
Number of patients	195	97	98
Median	1.200	1.200	1.150
Range	0.00–10.40	0.00–10.40	0.00–5.13
Tumor marker/CA15-3 (ng/mL)			
Number of patients	195	97	98
Median	7.80	7.60	8.00
Range	0.00–44.4	0.00–34.0	0.00–44.4

*BMI* body mass index, *CA15-3* Cancer antigen15-3, *CEA* carcinoembryonic antigen, *ER* estrogen receptor, *FSH* follicle-stimulating hormone, *LH* luteinizing hormone, *PgR* progesterone receptor, *SD* standard deviation

The majority of patients in the mFAS had good medication compliance with the study treatment: 97 (98.0%) patients in the 2-year group and 97 (95.1%) patients in the ≥ 3-year group received 8 and ≥ 12 doses of leuprorelin as specified in the protocol, respectively. Among the 102 patients in the ≥ 3-year group, 16 patients (15.7%) received 12 doses, 5 patients (4.0%) received 13–19 doses, and 76 patients (74.5%) received 20 doses, the maximum dose in the study. Each group had good compliance with tamoxifen treatment throughout the 5-year administration period. These compliances were similar to those reported in the FAS [24].

### Primary outcome

**Fig. 3:**
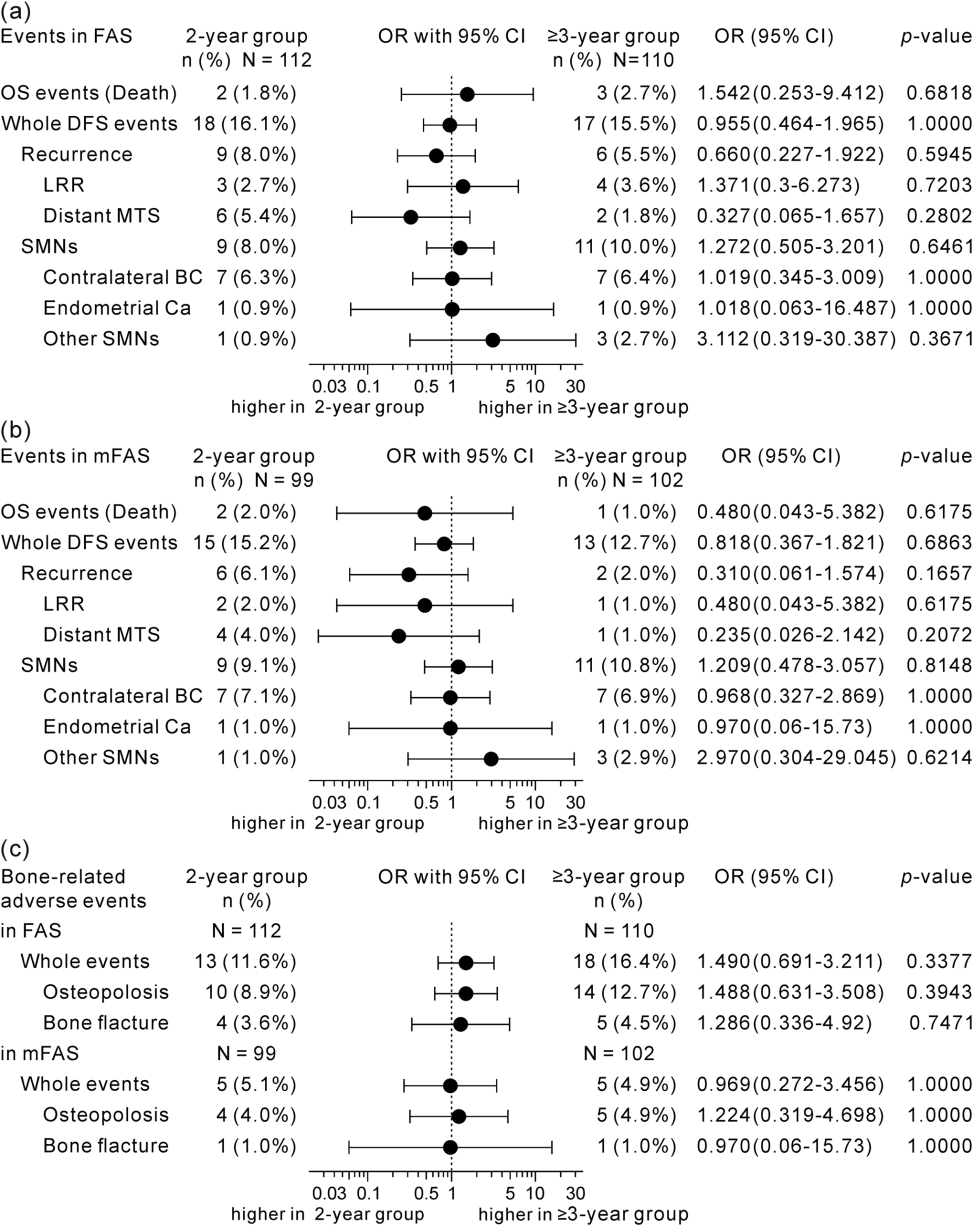
OR comparing incidence rates of OS, DFS and bone-related adverse events in 3-or-more-year treatment group with those in 2-year treatment group. OR of incidences of death and DFS events in patients treated with leuprorelin for 3 or more years versus those treated with it for 2 years **a** in FAS and **b** in mFAS. **c** OR of incidences of bone-related adverse events between these two groups. *P* value was calculated using 2-sided Fisher’s exact test. *Other SMNs* esophageal carcinoma (*n* = 1 in 2-year treatment group), and rectal carcinoma, cervical carcinoma and cutaneous lymphoma (each *n* = 1 in ≥ 3-year treatment group), *modified FAS* dataset from patients who were available to continue the study from 2 years (96 weeks) after the randomization. *CI* confidence interval, *DFS* disease-free survival, *FAS* full analysis set, *LRR* local–regional recurrence, *mFAS* modified FAS, *MTS* metastasis, *OR* odds ratio, *OS* overall survival, *SMNs* Secondary malignant neoplasms

DFS events (local–regional or distant recurrences and secondary malignancies) are summarized in Fig. 3a, b. Throughout the 10-year study period after randomization in the FAS, there were 35 disease events (18 and 17 in the 2- and ≥ 3-year groups, respectively). The fraction of recurrences, especially distant metastases showed lower trend in the ≥ 3-year group (1.8% and 1.0% in FAS and mFAS, respectively) compared with that in the 2-year group (5.4% and 4.0%), though not significant.

**Fig. 4:**
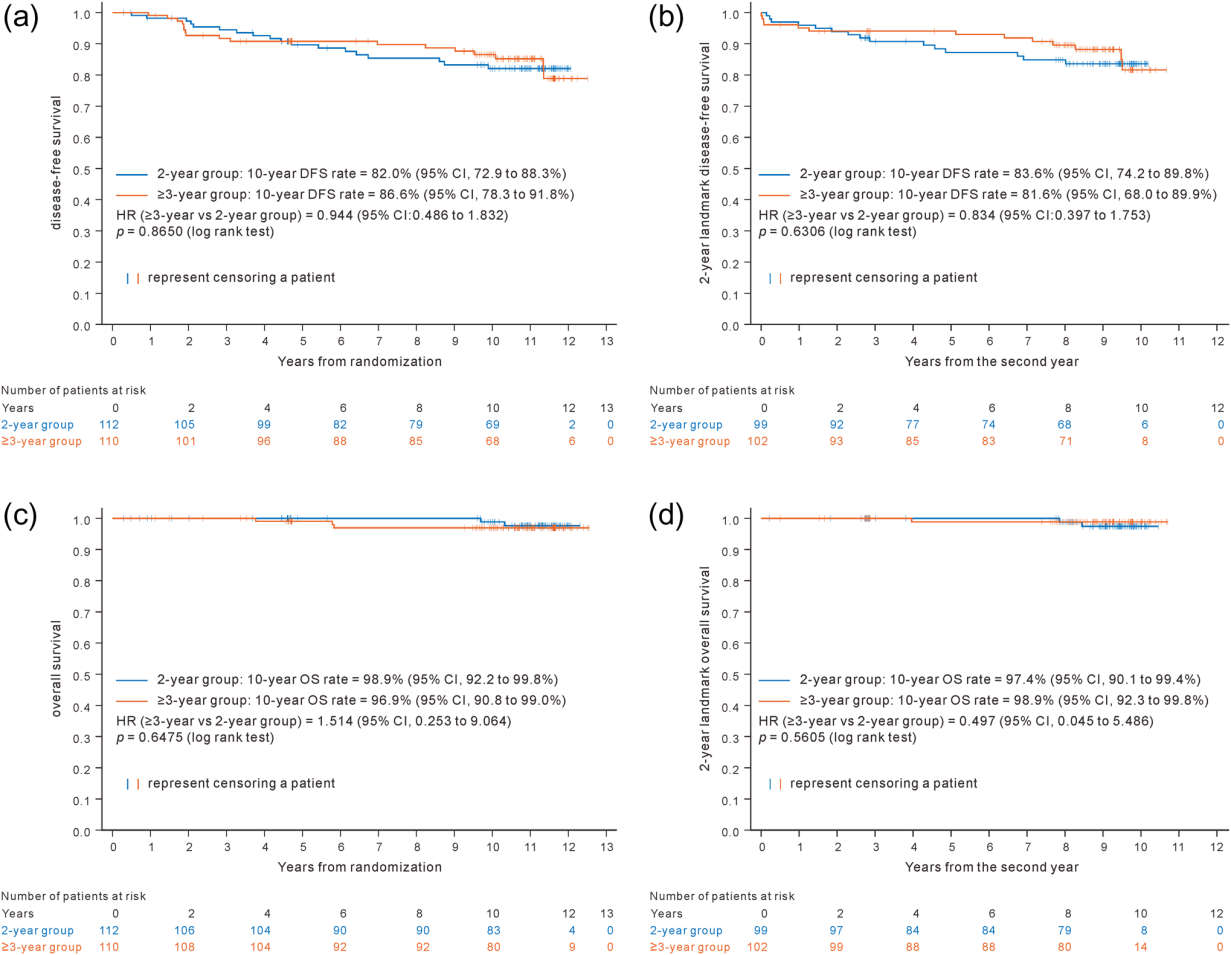
DFS and OS compared between 2-year and 3-or-more-year treatment groups in FAS and mFAS. **a** DFS in FAS. **b** 2-year landmark DFS in mFAS. **c** OS in FAS. **d** 2-year landmark OS in mFAS. 2-year landmark DFS/OS, defined as the time from the second year after randomization to DFS or OS events, *modified FAS* dataset from patients who were available to continue the study from 2 years (96 weeks) after the randomization. *DFS* disease-free survival, *FAS* full analysis set, *mFAS* modified FAS, *OS* overall survival

Figure 4a shows the Kaplan–Meier curves of DFS for patients in the FAS. There were also no significant differences between the two groups (HR 0.944 [95% CI 0.482–1.832]; log rank test, *p* = 0.865). Figure 4b shows the Kaplan–Meier analysis of DFS_L2y analyzed using the mFAS. HR (≥ 3-year versus 2-year group) was 0.834 (95% CI 0.397–1.753) and the 10-year DFS rate was 83.6% (95% CI 74.2–89.8%) and 81.6% (68.0–89.9%) in the 2- and ≥ 3-year groups, respectively. No significant differences were detected between the two groups in FAS and mFAS.

### Secondary outcomes

OS events are shown in Fig. 3a, b. One patient in the ≥ 3-year group of the FAS who died in a natural disaster (earthquake) was censored at the time of death. There were 5 and 3 cases of death in the FAS and mFAS, respectively. The number of events was too small to evaluate the difference of survival probabilities between the two treatment groups. Figure 4c, d shows the Kaplan–Meier analyses of OS for patients in the FAS and mFAS, respectively. There were no significant differences between the two groups.

### Subgroup analysis of DFS in different risk groups

Additional survival analysis was performed in subgroups classified into higher-risk and low-risk patients based on the St.Gallen International Consensus Guidelines for the systemic therapy of early breast cancer 2019 with some modifications adapting the present study [18]. The guidelines recommend the OFS with tamoxifen/AI for stage III and node-positive stage II patients, and also for node-negative stage II patients aged < 35 years, with large T or high grade and/or adverse gene signatures as the higher risk patients.

**Fig. 5:**
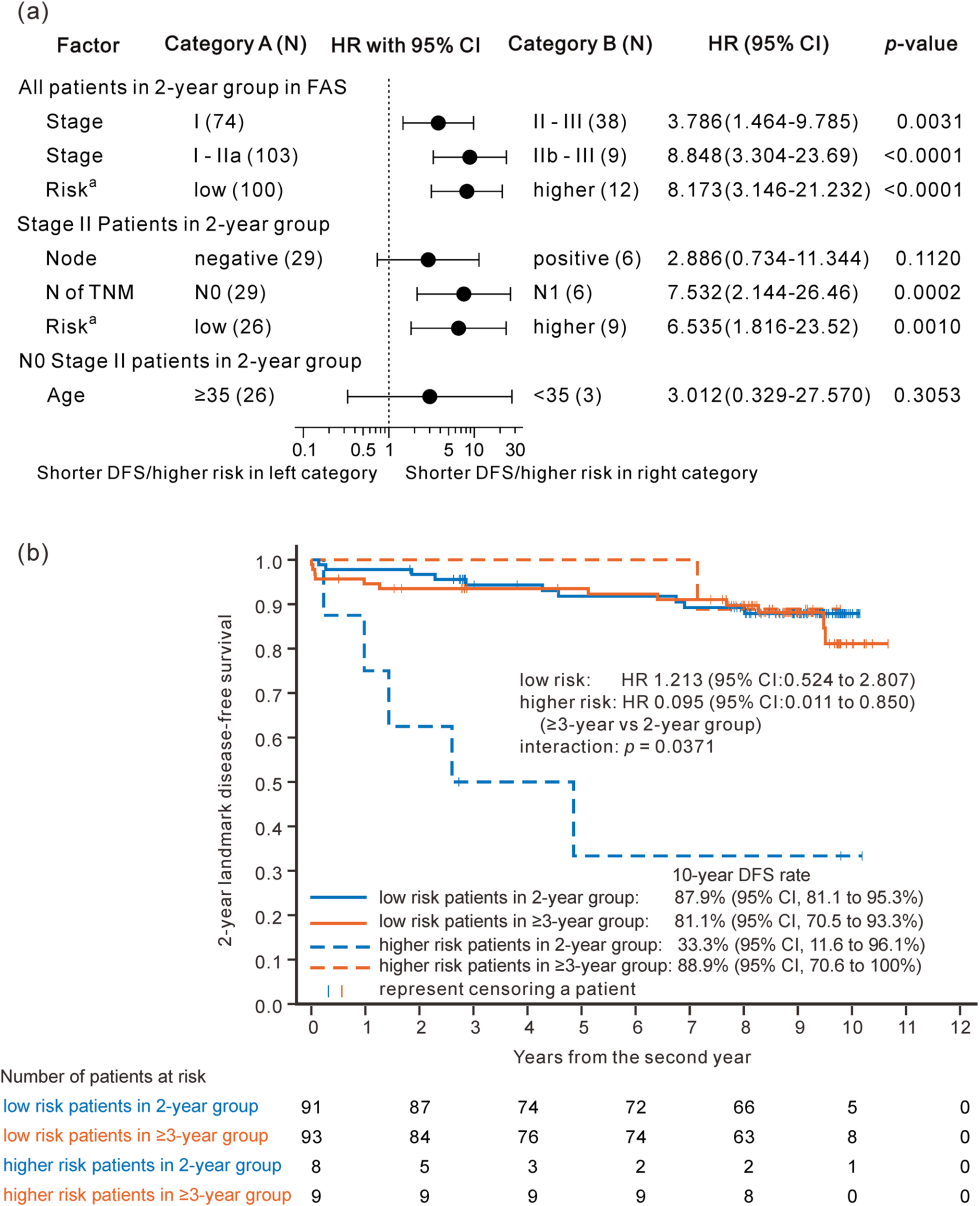
2-year landmark DFS compared between 2-year and 3-or-more-year treatment groups in higher- and low-risk patient subgroups. **a** DFS subgroup analysis to verify clinical risk factors in 2-year treatment groups using univariate Cox regression analysis. *P *value was determined by a two-sided log-rank test. ^a^Risk was classified into low- and higher-risk based on the breast cancer stage considering lymph node status and patient age as shown below. **b** 2-year landmark DFS, defined as the time from the second year after randomization to DFS events. The interaction between treatment (3-or-more versus 2-year treatment) and risk level (higher and low) was analysed using the multivariate Cox proportional-hazard model. Interaction *p*-value was determined using a 2-sided Wald test. *Higher-risk patients* patients who had ≥ stage IIb or who were aged under 35 years in stage IIa, *low-risk patients* patients who had stage I or who were aged ≥ 35 in stage IIa, *modified FAS* dataset from patients who were available to continue the study from 2 years (96 weeks) after the randomization. *DFS* disease-free survival, *mFAS* modified full analysis set

We evaluated the association of these clinical factors with DFS risk retrospectively by the subgroup analysis in 2-year group (*n* = 112) using univariate Cox model. No significant difference of DFS was seen between node-positive and negative stage II patients. On the other hand, it was significantly shorter in N1 stage II patients, all of whom had T2N1 classified into stage IIb than that in N0 stage II patients (stage IIa) (Fig. 5a). The DFS was highly significantly shorter in stage IIb and III patients compared with stage I and IIa patients. No large T (T3) was seen in stage II. Gene signature data were not available in this study. Although no significant difference was observed in DFS between N0 stage II patients aged < 35 and ≥ 35 years in small sample size, HR (age < 35 versus  ≥ 35) was 3.0 (shorter DFS in younger patients). In consequence, stage IIa aged ≥ 35 and stage I patients were classified into low-risk group, and stage IIa aged < 35 and stage IIb-III patients were defined as higher-risk group in this study.

Kaplan–Meier analysis and Cox regression of DFS_L2y was performed in the subgroups of higher- and low-risk patients according to the criteria as mentioned above in the mFAS (Fig. 5b). While no significant difference of DFS_L2y was observed between the two treatment groups in low-risk subgroup, DFS_L2y in higher-risk subgroup was better in the ≥ 3-year group (HR 0.095 [95% CI 0.011–0.850]) than that in the 2-year group, where interaction of the treatment by risk subgroup was significant with *p* = 0.0371. 10-year survival rates of DFS_L2y were 87.9, 81.1, and 88.9% in 2-year and ≥ 3-year groups in low-risk subgroup, and ≥ 3-year group in higher-risk subgroup, respectively, but 33.3% in 2-year group in higher-risk subgroup. The efficacy of 2-year treatment seems insufficient for higher-risk patients.

### Bone-related adverse events

Throughout the study period, the incidence of patients with bone-related AEs (osteoporosis or osteopenia, and bone fracture) was 11.6% (13/112) and 16.4% (18/110) in the 2- and ≥ 3-year groups, respectively, in the FAS (Fig. 3c). However, they were decreased to 5.1% (5/99) and 4.9% (5/102) in the mFAS after the second year (Fig. 3c). This means that the incidences of bone-related AEs were lower 2 years after the randomization and they were not different between the two groups, even though the administration of leuprorelin was continued for another 1–3 years only in the ≥ 3-year group.

### Metastasis and OS

There were only 5 and 3 cases of death (OS events) reported in the FAS and mFAS, respectively (Fig. 3a, b). Since DFS is considered as a surrogate endpoint of OS, outcomes (death) of patients who developed diseases (DFS events) are summarized in Table 2. Distant metastases appeared to be associated with death; namely, 37.5% (3/8) of patients who developed metastases died from the cancer. Although the number of cases was small, this fraction of death cases seems higher compared with death in 14.3% (1/7) of patients with local–regional recurrences or death in 5.0% (1/20) of those who developed secondary malignancies. Three other patients were reported to have developed metastases as a second disease after the first DFS events (local–regional recurrence and secondary malignancy) and 2 of them died. In all, 11 patients developed metastases (first DFS events, 8; second diseases, 3) and 5 patients among them died from cancer (45.5%). The most frequent metastatic site was bone (8/11 patients), followed by liver (3/11) and lung (2/11), allowing duplication of patients with multi-site metastases.

**Table 2:** Outcomes (death) of patients who developed DFS events in FAS population

Events	Number of patients with disease events	Outcomes (death) *n* (%) [95% CI]
Total death events in FAS (*N* = 222)		5
First diseases (DFS events)	35	5 (14.3%) [4.8–30.3%]
Recurrences	15	4 (26.7%) [7.8–55.1%]
Local–regional recurrence	7	1 (14.3%) [0.4–57.9%]
Distant metastasis	8	3 (37.5%) [8.5–75.5%]
Secondary malignancy	20	1 (5.0%) [0.1–24.9%]
Second diseases reported^a^	4	3 (75.0%) [19.4–99.4%]
Distant metastases	4	3 (75.0%) [19.4–99.4%]
After the 1st distant metastasis	1	1 (100%) [2.5%-NE]
After local–regional recurrence	1	1 (100%) [2.5%-NE]
After secondary malignancy	2	1 (50.0%) [1.3–98.7%]
All patients with distant metastases	11	5 (45.5%) [16.7–76.6%]

^a^Second diseases among all available data reported in patients who experienced the first diseases (DFS events)

*CI* confidence interval, *DFS* disease-free survival, *FAS* full analysis set, *NE* not estimable

## Discussion

This study could not demonstrate significant difference between adjuvant leuprorelin treatments for ≥ 3 (up to 5) years and 2 years concomitantly with tamoxifen in DFS and OS throughout 10-year study period in either FAS or mFAS (Fig. 4). This follow-up study was conducted continuing the RCT planed as a pilot study without sufficient statistical power, where 5-year DFS rate was estimated at 70 and 75% in 2-year and ≥ 3-year groups, respectively [24], and total sample size required was 2500 for 80% power at two-sided 5% significance level. If the DFS time simply follows exponential distribution, 10-year DFS rate is anticipated at 49% and 56% in these groups and improvement of statistical power is expected. In the results obtained from this study, however, the 10-year DFS rate was 81.6% and 83.6% in 2-year and ≥ 3-year treatment group, respectively. The high DFS rate in this study is thought to be due to an improvement in total management of breast cancer and high fraction of low-risk patients enrolled (95.1% of ≤ stage IIa).

OS is the most reliable endpoint in breast-cancer studies [33]. DFS is a kind of surrogate endpoint for OS, but DFS events are not necessarily associated with death. Distant metastases in DFS events are known to cause disability and death in patients with breast cancer. Distant recurrence-free survival is recommended as a surrogate endpoint to provide early indication of OS results [33]. Our results seem consistent with these opinions, which suggests the association of distant metastasis with death (Table 2). DFS is often used when expecting to obtain the results earlier and with a smaller sample size compared to the OS study. For the endocrine treatment, however, some of the DFS events, particularly some kinds of secondary malignancies, are not connected with the mode of action of leuprorelin, different from cytotoxic chemotherapy. In our study, secondary malignancies were identified in 20 out of 35 patients (57.1%) with DFS events (Table 2 and Fig. 3a, b). The most frequent disease was second primary contralateral breast cancer (*n* = 14); however, lymphoma and rectal, oesophageal, and cervical cancers were reported as the DFS events of second malignancies (Fig. 3a, b). It is probable that LH-RH agonists could not suppress their development. Additionally, secondary malignancies were less associated with death in this study (Table 2). DFS might not necessarily be appropriate as the endpoint to evaluate the efficacy of leuprorelin in this long-term study in which participants had a risk of developing secondary cancers spontaneously and independently of study drugs, though this risk was expected to be compensated by the same events occurring in the comparator arm.

The subgroup analysis showed that higher-risk patients treated with leuprorelin for ≥ 3 years had significantly longer DFS compared with those treated for 2 years, while no difference was seen between the treatments in patients with low risk (Fig. 5b). Although the sample size of higher-risk subgroup was as small as 17 patients, the result is consistent with recent guidelines, which recommend combination with 5-year OFS and tamoxifen or aromatase inhibitor (AI) for higher-risk premenopausal patients as an endocrine adjuvant therapy for resected early stage breast cancer [18]. However, the guidelines recommend tamoxifen or AI monotherapy without OFS for low-risk patients [18]. The recommendations are based on the data reported from an 8-year update of the SOFT trial of OFS for premenopausal women with resected breast cancer [16]. The report concluded that the addition of OFS to tamoxifen for 5 years resulted in significantly higher 8-year rates of both DFS and OS than tamoxifen alone, but the frequency of AEs was higher in the combination groups with OFS than in the tamoxifen-alone group. Our results can be interpreted as showing that addition of 5-year tamoxifen to 2-year treatment with leuprorelin was sufficient for low-risk patients but not sufficient for higher-risk patients, in which the combination effect of longer treatment with leuprorelin was demonstrated.

The SOFT study demonstrated that 5-year combination of tamoxifen and OFS is superior to tamoxifen alone, but treatment duration of OFS has not been established yet [16]. SOFT also recommended tamoxifen or AI alone rather than their combination with OFS for the low-risk patients from the viewpoint of risk and benefit, but risk and benefit of the combination with 2-year OFS and tamoxifen has not been fully evaluated in comparison with that of tamoxifen alone, particularly in Japanese patients. The present study can provide useful information to answer these questions.

In the previous RCT, the incidence of treatment-related AEs was significantly higher in the ≥ 3-year group than in the 2-year group, but the most treatment-related AEs were of grades 1 and 2 during 5 years after the randomization [24]. The increase in the treatment duration of leuprorelin led to a decrease in the BMD, but there were neither increases in the severity of AEs nor occurrences of any new types of AEs [24]. Our present follow-up study demonstrated the safety after the long-term administration of leuprorelin plus tamoxifen up to 5 years with 10-year follow-up period. No difference was seen in bone-related AEs between 2-year and ≥ 3-year groups (70% treated for 5 years). This suggested that most bone-related AEs that develop during leuprorelin treatment can be recovered after its cessation. This safety profile may help in the decision on the indication for 5-year administration of leuprorelin plus tamoxifen to treat Japanese breast cancer. Recently, the long-term treatment with OFS has not been recommended for low-risk breast cancer patients from the viewpoint of risk and benefit [18]. Combining our studies, the risks of long-term leuprorelin are not so high, and similar to those of previous standard treatment for 2 years in Japanese patients. More investigation is needed in consideration of racial or ethnic differences.

This study had some limitations. First, a difference in primary endpoint of DFS was not detected between 2-year and ≥ 3-year groups, but this does not mean that 2 years is the appropriate treatment duration of leuprorelin. This null result is due to the low statistical power caused by small sample size, which arose from overestimation of number of events and/or higher fraction of low-risk patients enrolled than our expectation. We could not clarify the primary objective of appropriate treatment duration. However, exploratory subgroup analysis suggested that ≥ 3-year treatment (mostly 5-year) is appropriate for the higher-risk patients, though the sample size was small. Second, 6 and 13 patients withdrew their consent and terminated the study early in 2-year group, while 0 and 4 terminated the study early in ≥ 3-year group before 2 years after randomization and throughout the study, respectively (Fig. 2). Some reasons were reported in the 2-year group; 1 patient required more treatment with leuprorelin at the end of planned 2-year treatment, and 3 patients desired retreatment after the menses resumed. Some bias was inevitable in this comparison study between different treatment durations of leuprorelin.

Although this study did not have sufficient statistical power to clarify the difference between the DFS in the two groups, adjuvant leuprorelin treatment for ≥ 3 years with tamoxifen did not show significant difference in DFS with that for 2 years among all patients with premenopausal endocrine-responsive breast cancer in FAS and mFAS up to the 10th year after the randomization, but suggested greater benefit in higher-risk patients. Leuprorelin treatment for ≥ 3 years showed similar safety profiles to those for 2 years without new safety signals.
